# The IDEAL classification system: a new method for classifying fractures of the distal extremity of the radius - description and reproducibility

**DOI:** 10.1590/1516-3180.2013.1314496

**Published:** 2013-08-01

**Authors:** João Carlos Belloti, João Baptista Gomes dos Santos, Vinícius Ynoe de Moraes, Felipe Vitiello Wink, Marcel Jun Sugawara Tamaoki, Flávio Faloppa

**Affiliations:** I MD, PhD. Hand Surgeon and Professor, Discipline of Hand Surgery, Universidade Federal de São Paulo/Escola Paulista de Medicina (Unifesp/EPM), São Paulo, Brazil.; II MD. Hand Surgery Resident, Universidade Federal de São Paulo/Escola Paulista de Medicina (Unifesp/EPM), São Paulo, Brazil.; III MD. Hand Surgeon, Discipline of Hand Surgery, Universidade Federal de São Paulo/Escola Paulista de Medicina (Unifesp/EPM), São Paulo, Brazil.; IV MD, PhD. Orthopedic Surgeon, Discipline of Hand Surgery, Universidade Federal de São Paulo/Escola Paulista de Medicina, (Unifesp/EPM), São Paulo, Brazil.; V MD, PhD. Hand Surgeon, Full Professor, Discipline of Hand Surgery, Universidade Federal de São Paulo/Escola Paulista de Medicina (Unifesp/EPM), São Paulo, Brazil.

**Keywords:** Colles’ fracture, Classification, Wrist, Radius fractures, Reproducibility of results, Fratura de Colles, Classificação, Punho, Fraturas do rádio, Reprodutibilidade dos testes

## Abstract

**CONTEXT AND OBJECTIVE:**

There is no consensus concerning which classification for distal radius fractures is best and the existing methods present poor reproducibility. This study aimed to describe and assess the reproducibility of the new IDEAL classification, and to compare it with widely used systems.

**DESIGN AND SETTING:**

Reproducibility study, Hand Surgery Section, Universidade Federal de São Paulo.

**METHODS:**

The IDEAL classification and its evidence-based rationale are presented. Sixty radiographs (posteroanterior and lateral) from patients with distal radius fractures were classified by six examiners: a hand surgery specialist, a hand surgery resident, an orthopedic generalist, an orthopedic resident and two medical students. Each of them independently assessed the radiographs at three different times. We compared the intra and interobserver concordance of the IDEAL, AO, Frykman and Fernandez classifications using Cohen’s kappa (κ) (for two observers) and Fleiss’s κ (for more than two observers).

**RESULTS:**

The concordance was high for the IDEAL classification (κ = 0.771) and moderate for Frykman (κ = 0.556), Fernandez (κ = 0.671) and AO (κ = 0.650). The interobserver agreement was moderate for the IDEAL classification (κ = 0.595), but unsatisfactory for Frykman (κ = 0.344), Fernandez (κ = 0.496) and AO (κ = 0.343).

**CONCLUSION:**

The reproducibility of the IDEAL classification was better than that of the other systems analyzed, thus making the IDEAL system suitable for application. Complementary studies will confirm whether this classification system makes adequate predictions for therapy and prognosis.

## RESUMO

**CONTEXTO E OBJETIVO:**

Não existe consenso sobre qual é a melhor classificação para as fraturas do rádio distal e os métodos existentes apresentam baixa reprodutibilidade. Este estudo tem como objetivo descrever e avaliar a reprodutibilidade de uma nova classificação (a IDEAL) comparando-a com as classificações mais amplamente utilizadas.

**TIPO DE ESTUDO E LOCAL:**

Estudo de reprodutibilidade, Disciplina de Cirurgia da Mão, Universidade Federal de São Paulo.

**MÉTODOS:**

Apresentamos a classificação IDEAL e sua fundamentação teórica baseada em evidências. Sessenta radiografias (anteroposterior e de perfil) de pacientes com fraturas do rádio distal foram classificadas por seis examinadores: um especialista e um residente de cirurgia da mão, um ortopedista, um residente de ortopedia e dois estudantes de medicina. Cada um, independentemente, avaliou as radiografias em três momentos diferentes. Analisamos a concordância intra e interobservador da classificação IDEAL, AO, Frykman e Fernandez, utilizando o kappa (κ) de Cohen (para dois observadores) e κ de Fleiss (para mais de dois observadores).

**RESULTADOS:**

A concordância demonstrou-se elevada para a classificação IDEAL (κ = 0,771) e moderada para Frykman (κ = 0,556), Fernandez (κ = 0,671) e AO (κ = 0,650). A concordância interobservador foi moderada para a classificação IDEAL (κ = 0,595), mas insatisfatória para Frykman (κ = 0,344), Fernandez (κ = 0,496) e AO (κ = 0,343).

**CONCLUSÃO:**

A reprodutibilidade da classificação IDEAL se demonstrou superior quando comparada às analisadas neste estudo, tornando a classificação IDEAL adequada para aplicação. Estudos complementares confirmarão se esta classificação é adequada para previsão de terapia e prognóstico.

## INTRODUCTION

Distal radius fractures occur in approximately one in every 10,000 people, accounting for 16% of skeletal fractures and 74% of forearm fractures.^1^^,^^2^^,^^3^ The most common mechanism of injury is a fall on the hand with hyperextension. The fracture characteristics (location, joint involvement, degree of comminution and soft tissue injury) are directly related to the trauma energy, position of the hand at the moment of trauma and bone quality.^2^ Appropriate fracture treatment requires a good understanding of these fracture characteristics.^4^

Fracture classification systems were developed to divide fractures into different types and consequently serve as a guide to treatment. Starting more than a century ago, Colles, Smith, Barton, Pouteau, Goyrand and others described the morphology of fractures for use in classification.^2^^,^^5^^,^^6^^,^^7^ The advent of radiology enabled a more accurate analysis of fracture characteristics, including the degree of displacement and presence of joint fracture. Lie-Nielsen in 1939^8^ and Gartland and Werley in 1951^9^ based their classification systems on the presence or absence of intra-articular involvement, metaphyseal comminution and/or deformity; however, neither system evaluated fragment displacement. In 1959, Lindstrom expanded these criteria into six groups, describing fragment displacement and joint involvement in detail.^10^ In 1967, Frykman established a classification system that took into account involvement of the radiocarpal joint, distal radioulnar joint and ulnar styloid.^11^ The AO classification, which was created in 1986 and revised in 1990, determines the seriousness of the fracture according to joint involvement and metaphyseal comminution. The AO system is comprehensive, but its intraobserver and interobserver reproducibility are not high.^12^^,^^13^ Another classification, proposed by Fernandez, is based on the mechanism of trauma.^14^ This classification was designed to be practical, predict stability, identify equivalent lesions in children and provide general recommendations for treatment.

An effective classification system must be valid, reliable and reproducible, but it should also standardize a language for consistent communication, provide guidelines for appropriate treatment, indicate the likelihood of complications and fracture instability and predict a realistic prognosis for each fracture.^15^ The system should also provide a mechanism for evaluating and comparing treatment results with results from similar fractures in different centers reported at different times.^16^^,^^17^

Currently, none of the classification systems available have reproducibility that adequately provides evidence for treatment and prognosis.^13^^,^^15^^,^^18^^,^^19^

## OBJECTIVES

In this study, we aimed to describe a new classification method for distal radius fractures, which we named the “IDEAL Classification”, assess its reproducibility (intraobserver and interobserver agreement) and compare IDEAL with established classification systems (AO, Frykman and Fernandez classification systems)

## METHODS

This study was approved by our institution’s ethics committee (reference: 1225/10).

### Description of IDEAL classification

The IDEAL fracture classification is based on the epidemiological and radiographic factors that are important for treatment and prognosis. Using this method, we classified the fracture during the first patient evaluation using two epidemiological criteria (patient age and trauma energy) and three radiographic parameters assessed at the initial radiographic examination (posteroanterior and lateral): displacement of the fragments, joint incongruity and associated injuries (Table 1).

**Table 1.** IDEAL classification system: rationale and scoring

	Parameter	0 points	1 point
I	Joint incongruity	No	Step or gap > 2 mm
D	Displacemen	No	Requires reduction
E	Energy^1^	Low	High
A	Age	< 60 years old	³ 60 years old
L	Associated lesions^2^	Absent	Present

^1^Low = fall from standing height, or High = other; ^2^Open fracture/carpal fractures and/or instability/distal ulnar fractures.

Each of the five characteristics is given a score of zero or one (total score, 0-5 points). The criteria used to determine the IDEAL score are as follows:

1. Incongruity: joint step or gap ³ 2 mm (1 point); or < 2 mm (0 points);
2. Displacement: radial shortening > 3 mm, loss of volar tilt > 10^o^ or loss of radial inclination > 5^o^ (1 point); minimal or no displacement (0 points);
3. Energy: high-energy, e.g. fall from a height or traffic accident (1 point); or low-energy, e.g. fall from standing height (0 points);
4. Age: > 60 years (1 point); or < 60 years (0 points);
5. Lesions: e.g. radiocarpal dislocation or subluxation, carpal bone fracture, carpal or distal radioulnar instability or neurovascular injuries) (1 point); no lesions (0 points).

After scoring the five characteristics, the fractures can be classified into one of three fracture types according to severity and complexity (Table 2).

**Table 2.** IDEAL classification system: description and treatment/prognosis guidance

Type	Score	Description	Treatment	Prognosis
I	0-1 points	Stable	Conservative	Good
II	2-3 points	Potentially unstable	Pins/external fixation/plating	Intermediate
III	4-5 points	Complex	Associated methods/bone graft	Poor

Type I fractures (0-1 points) are potentially stable. These are fractures in the elderly without displacement or displaced fractures in young patients, caused by low-energy trauma, without joint incongruity or associated injuries. They are generally treated conservatively with closed reduction and a cast and have a good prognosis.

Type II fractures (2-3 points) are potentially unstable fractures with displacement that have a high potential for loss of reduction and malunion because of poor bone quality (elderly patients) and/or associated high-energy trauma, joint incongruity or correlated injuries (both young and elderly patients). Type II fractures require surgical stabilization, such as percutaneous pinning, external fixation or internal fixation with plates. These fractures are more susceptible to the potential complications inherent to surgery. The prognosis depends on the success of surgery.

Type III fractures (4-5 points) are complex fractures with displacement. They are usually caused by high-energy trauma and are associated with joint incongruity and related injuries. Because of their inherent instability and potential irreducibility, they often require open reduction, associated methods of fixation and, possibly, bone grafting. They are prone to complications and have a poor prognosis, regardless of the treatment method.

### Evaluation of reproducibility

Between November 2010 and May 2011, a convenience sample of 60 adult patients treated at institution X was included in this study. We collected distal radius fracture radiographs, which were analyzed and classified using the IDEAL, AO, Frykman and Fernandez classification systems by six observers with different degrees of experience: a hand surgeon with more than 20 years of experience, a general orthopedic surgeon, a medical resident in hand surgery, a medical resident in orthopedics and traumatology and two medical students. All the assessments were performed prospectively.

The AO classification describes fractures in alphanumeric terms. It mainly divides fracture types according to the presence (B/C group) or the absence (A group) of joint fracture. Its main subgroup relates to specific fracture patterns, ranging from A1 to C3. The Frykman classification is based on a description of eight categories. It takes ulnar/distal radioulnar involvement into consideration and uses this as a differential. The Fernandez classification is based on trauma mechanism and encompasses five categories, in which the last category includes combined mechanisms.

The radiographs were digitized and numbered sequentially. All identification was then concealed, and the radiographs were randomized by an unique sequence of numbers generated by a computer program. Randomization was performed by a researcher not involved in the assessments. The radiographs were assessed three times (T1, T2 and T3) in a random sequence by each observer, with two-week intervals between each of the assessments.

### Statistical methods

To determine the intraobserver reproducibility, we used the method proposed by Fleiss et al.,^20^ which calculates the degree of agreement in relation to what would be expected by chance. We also used this method to determine the interobserver agreement between more than two observers. Cohen’s kappa (k) coefficient was used to determine the inter-rater agreement of two observers. In general, k values less than 0.5 are considered unsatisfactory, values between 0.5 and 0.75 are considered satisfactory, and values greater than 0.75 are considered excellent.^21^^,^^22^

Comparison of the intraobserver reproducibility between the first two evaluations (T1 versus T2) with the intraobserver reproducibility between the second two evaluations (T2 versus T3) revealed the effect of conditioning on the classification. We used the method of Giraudeau and Mary to determine sample size according to the expected intraobserver agreement and confidence interval. For an expected k of 0.70 and confidence interval of 90%, 50 samples would be needed.^17^

The study results were not influenced by the external funding source and this source did not play any role in the investigation.

## RESULTS

Six different observers each evaluated 60 radiographs on three different occasions using the four classification systems, to produce a total of 4320 ratings during the study. The intraobserver reproducibility of the three assessments (T1, T2 and T3) was excellent for the IDEAL classification (k = 0.771) and satisfactory for the Frykman (k = 0.556), Fernandez (k = 0.671) and AO (k = 0.650) classifications (Table 3). When the reproducibility of the first two observations (T1 versus T2) was compared with the reproducibility of the last two (T2 versus T3), the results were similar without substantial improvement in the level of agreement.

**Table 3.** Intraobserver reproducibility (T1, T2, T3) of the fracture classification

	IDEAL	Frykman	Fernandez	AO
Hand surgery resident	0.794	0.604	0.734	0.740
Hand surgery specialist	0.882	0.682	0.720	0.707
General orthopedic surgeon	0.906	0.677	0.737	0.680
Orthopedics resident	0.764	0.596	0.768	0.720
Medical student 1	0.497	0.336	0.456	0.463
Medical student 2	0.804	0.491	0.612	0.592
Mean	0.771	0.556	0.671	0.650

SE = standard error of the mean; T1 = first assessment; T2 = second assessment; T3 = third assessment.

As shown in Table 4, the interobserver agreement for the three observations was satisfactory for the IDEAL classification (k = 0.595), but unsatisfactory for the other classifications, with the AO classification showing the worst agreement (k = 0.343). The average concordance between observer pairs for the IDEAL classification was satisfactory at all three time points (T1: k = 0.653; T2: k = 0.595; and T3: k = 0.537) and for the Fernandez classification on two occasions (T1: k = 0.515; and T2: k = 0.534), but it was unsatisfactory for the AO and Frykman classifications at all times.

**Table 4.** Interobserver reproducibility (T1, T2, and T3) of the fracture classification systems

	IDEAL	Frykman	Fernandez	AO
First assessment (T1)	0.654	0.328	0.509	0.316
Second assessment (T2)	0.594	0.391	0.533	0.393
Third assessment (T3)	0.537	0.314	0.447	0.319
Mean	0.595	0.344	0.496	0.343

The mean intraobserver agreement of the IDEAL classification was significantly higher than that of the Frykman classification (Table 5). Similarly, the mean interobserver agreement of the IDEAL classification was significantly higher than those of the AO and Frykman classifications and was similar to that of the Fernandez classification (Table 5).

**Table 5.** Comparison of intraobserver and interobserver κ values according to classification system

Classification systems	Intraobserver agreement P-value	Interobserver agreement P-value
IDEAL, Frykman, Fernandez and AO	0.06	0.04
IDEAL versus Frykman	0.02	0.03
IDEAL versus Fernandez	0.21	0.82
IDEAL versus AO	0.13	0.03
Frykman versus Fernandez	0.17	0.02
Frykman versus AO	0.25	0.96
Fernandez versus AO	0.74	0.01

Fisher F-test.

## DISCUSSION

The current classification systems for distal radial fractures are based on fracture morphology^11^^,^^12^^,^^23^ or mechanism of injury.^14^^,^^24^ In the IDEAL classification, we aimed to provide data that would be relevant for treatment guidance and prognosis, by using five key elements: two epidemiological factors (Age and trauma Energy) and three radiographic factors (Displacement, joint Incongruity, and associated Lesions).

An evidence-based rationale was used in order to develop this classification. When planning treatment, advanced patient age is one of the most important prognostic factors for instability,^25^^,^^26^ whereas low-energy fractures have less potential for instability.^25^^,^^26^ Fractures with joint incongruity > 2 mm or unacceptable angular or shortening displacement have higher morbidity and worse prognosis than shown by fractures with little or no displacement.^27^ Associated injuries such as carpal instability, unstable distal radioulnar joint lesions or carpal/radiocarpal ligament injury are also associated with worse prognosis^26^^,^^28^^,^^29^ and may require additional interventions.

We believe that a combination of these factors can guide proper planning of treatment. IDEAL is a mnemonic that is easy to remember and categorizes fractures into three main types, numbered according to seriousness and requirement for stabilization, which makes it feasible to apply this classification in clinical practice. Ilyas and Jupiter strengthened our classification rationale by stating that surgical indications can be placed into four categories: patient-related factors, fracture reduction, fracture stability and presence of associated injuries.^4^

A reliable distal radius fracture classification is necessary for systematic treatment of these fractures and is essential for comparing the results from different clinical studies.^12^^,^^13^^,^^15^^,^^19^^,^^31^^,^^32^ In the present study, the intraobserver and interobserver reproducibility of the IDEAL classification was generally higher than that of the established classifications. We believe that this classification system is more reliable because of clearness in assessing the classification features. The IDEAL classification system is easy to use and was reproducible, not only when used by the hand surgery specialist but also when used by the medical students.

The AO and Frykman classifications, and to some extent the Fernandez classification, aim to be comprehensive in describing fractures. However, the reliability of these systems is low, especially when subgroups are analyzed. van Leerdam et al.^13^ suggested more realistic goals for distal radius fracture classification systems: they should be simple, reproducible and clinically focused. We have attempted to follow this in developing the IDEAL classification.

In the present study, six evaluators with varying degrees of knowledge assessed radiographs according to the IDEAL system. They demonstrated that the level of expertise in evaluating fractures was not an important factor in relation to intraobserver reproducibility. In addition, no marked improvement in intraobserver reproducibility was seen between the second and third assessments. Thus, experience with the IDEAL classification system itself did not affect reproducibility, either.

The IDEAL classification is reliable and shows good reproducibility in comparison with the existing classification systems. Prospective studies are needed in order to verify its clinical effectiveness in predicting instability, planning treatments and making prognoses for these fractures.

## References

[1] Baron JA, Barrett JA, Karagas MR (1996). The epidemiology of peripheral fractures. Bone.

[2] Fernandez DL, Jupiter J (2002). Fractures of the distal radius.

[3] Róbertsson GO, Jónsson GT, Sigurjónsson K (1990). Epidemiology of distal radius fractures in Iceland in 1985. Acta Orthop Scand.

[4] Ilyas AM, Jupiter JB (2007). Distal radius fractures--classification of treatment and indications for surgery. Orthop Clin North Am.

[5] The classic (1972). On the fracture of the carpal extremity of the radius. Abraham Colles, Edinburgh Med Surg J., 1814. Clin Orthop Relat Res.

[6] Pouteau C. (1793). Oeuvres posthumes de M. Pouteau: memóire, contenant quelques réflexions sur quelques fractures de l’avant-bras, sur les luxations du incomplèttes poignet et sur lateral epicondylitis diastasis.

[7] Goyrand G (1832). Mémoire sur les fractures de l’extremité inférieure du radius qui simulent les luxations du poignet. Gazette de Medicine.

[8] Fernandez DL, Jupiter JB (1996). Fractures of the distal radius.

[9] Gartland JJ, Werley CW (1951). Evaluation of healed Colles’ fractures. J Bone Joint Surg Am.

[10] Lindstrom A (1959). Fractures of the distal end of the radius. A clinical and statistical study of end results. Acta Orthop Scand Suppl.

[11] Frykman G (1967). Fracture of the distal radius including sequelae--shoulder-hand-finger syndrome, disturbance in the distal radio-ulnar joint and impairment of nerve function. A clinical and experimental study. Acta Orthop Scand.

[12] Kreder HJ, Hanel DP, McKee M (1996). Consistency of AO fracture classification for the distal radius. J Bone Joint Surg Br.

[13] van Leerdam RH, Souer JS, Lindenhovius AL, Ring DC (2010). Agreement between Initial Classification and Subsequent Reclassification of Fractures of the Distal Radius in a Prospective Cohort Study. Hand (NY).

[14] Fernandez DL (2001). Distal radius fracture: the rationale of a classification. Chir Main.

[15] Belloti JC, Tamaoki MJ, Franciozi CE (2008). Are distal radius fracture classifications reproducible? Intra and interobserver agreement. Sao Paulo Med J.

[16] Martin JS, Marsh JL (1997). Current classification of fractures. Rationale and utility. Radiol Clin North Am.

[17] Karanicolas PJ, Bhandari M, Kreder H (2009). Evaluating agreement: conducting a reliability study. J Bone Joint Surg Am.

[18] Belloti JC, Santos JB, Atallah AN, Albertoni WM, Faloppa F (2007). Fractures of the distal radius (Colles’ fracture). Sao Paulo Med J.

[19] Ploegmakers JJ, Mader K, Pennig D, Verheyen CC (2007). Four distal radial fracture classification systems tested amongst a large panel of Dutch trauma surgeons. Injury.

[20] Fleiss JL, Cohen J, Everitt BS (1969). Large sample standard errors of kappa and weighted kappa. Psychological Bulletin.

[21] Landis JR, Koch GG (1977). The measurement of observer agreement for categorical data. Biometrics.

[22] Koch GG, Landis JR, Freeman JL, Freeman DH, Lehnen RC (1977). A general methodology for the analysis of experiments with repeated measurement of categorical data. Biometrics.

[23] Cooney WP (1993). Fractures of the distal radius. A modern treatment-based classification. Orthop Clin North Am.

[24] Fernández DL (1993). Fractures of the distal radius: operative treatment. Instr Course Lect.

[25] Mackenney PJ, McQueen MM, Elton R (2006). Prediction of instability in distal radial fractures. J Bone Joint Surg Am.

[26] Nesbitt KS, Failla JM, Les C (2004). Assessment of instability factors in adult distal radius fractures. J Hand Surg Am.

[27] Bradway JK, Amadio PC, Cooney WP (1989). Open reduction and internal fixation of displaced, comminuted intra-articular fractures of the distal end of the radius. J Bone Joint Surg Am.

[28] Lafontaine M, Delince P, Hardy D, Simons M (1989). L’instabilité des fractures de l’extrémité inférieure du radius: à propos d’une série de 167 cas. [Instability of fractures of the lower end of the radius: apropos of a series of 167 cases]. Acta Orthop Belg.

[29] Lafontaine M, Hardy D, Delince P (1989). Stability assessment of distal radius fractures. Injury.

[30] Belloti J, Moraes VY, Albers MB, Faloppa F, Dos Santos JB (2010). Does an ulnar styloid fracture interfere with the results of a distal radius fracture?. J Orthop Sci.

[31] Andersen DJ, Blair WF, Steyers CM (1996). Classification of distal radius fractures: an analysis of interobserver reliability and intraobserver reproducibility. J Hand Surg Am.

[32] Illarramendi A, González Della Valle A, Segal E (1998). Evaluation of simplified Frykman and AO classifications of fractures of the distal radius. Assessment of interobserver and intraobserver agreement. Int Orthop.

